# An Efficient Gene Excision System in Maize

**DOI:** 10.3389/fpls.2020.01298

**Published:** 2020-09-02

**Authors:** Ning Wang, Maren Arling, George Hoerster, Larisa Ryan, Emily Wu, Keith Lowe, William Gordon-Kamm, Todd J. Jones, N. Doane Chilcoat, Ajith Anand

**Affiliations:** Crop Genome Engineering, Applied Science and Technology, Corteva Agriscience, Johnston, IA, United States

**Keywords:** *Agrobacterium*, developmentally-regulated promoters, heat-shock promoters, morphogenic genes, marker-free events, rapid maize transformation

## Abstract

Use of the morphogenic genes *Baby Boom* (*Bbm*) and *Wuschel2* (*Wus2*), along with new ternary constructs, has increased the genotype range and the type of explants that can be used for maize transformation. Further optimizing the expression pattern for *Bbm*/*Wus2* has resulted in rapid maize transformation methods that are faster and applicable to a broader range of inbreds. However, expression of *Bbm*/*Wus2* can compromise the quality of regenerated plants, leading to sterility. We reasoned excising morphogenic genes after transformation but before regeneration would increase production of fertile T0 plants. We developed a method that uses an inducible site-specific recombinase (*Cre*) to excise morphogenic genes. The use of developmentally regulated promoters, such as *Ole*, *Glb1*, *End2*, and *Ltp2*, to drive *Cre* enabled excision of morphogenic genes in early embryo development and produced excised events at a rate of 25–100%. A different strategy utilizing an excision-activated selectable marker produced excised events at a rate of 53–68%; however, the transformation frequency was lower (13–50%). The use of inducible heat shock promoters (e.g. *Hsp17.7*, *Hsp26*) to express Cre, along with improvements in tissue culture conditions and construct design, resulted in high frequencies of T0 transformation (29–69%), excision (50–97%), usable quality events (4–15%), and few escapes (non-transgenic; 14–17%) in three elite maize inbreds. Transgenic events produced by this method are free of morphogenic and marker genes.

## Introduction

The use of the morphogenic genes *Bbm* and *Wus2* significantly increased transformation frequencies and reduced genotype dependence in many cereal crops (Lowe et al., 2016; Mookkan et al., 2017; Anand et al., 2018; Lowe et al., 2018). Morphogenic genes have enabled the development of a rapid transformation method involving direct formation of somatic embryos and T0 plants from immature scutella (Lowe et al., 2018). This approach has facilitated transformation (Lowe et al., 2016; Mookkan et al., 2017) and CRISPR/Cas-mediated editing (Chilcoat et al., 2017) in numerous elite maize inbreds, and enabled use of alternate explants, such as embryo slices from mature seeds or leaf segments (Lowe et al., 2016; Lowe et al., 2018). However, ectopic expression of the morphogenic genes causes pleiotropic effects including abnormal shoots/roots and infertile plants (Lowe et al., 2016). The use of promoters that drive high expression levels during the transformation process, but lower expression levels in the vegetative plant, somewhat ameliorates these problems (Lowe et al., 2018) but some negative effect and the presence of morphogenic genes is undesirable in commercial products. Transgenic plants regenerated through *de novo* meristem induction stimulated by morphogenic gene expression also showed developmental abnormalities (Maher et al., 2020), and without removal also raise concerns that non-visible pleiotropic effects are possible. Therefore, it is desirable to generate transgenic plants free from morphogenic genes. Previously, a method using a non-integrating *Wus2* gene approach recovered fertile T0 plants free-off morphogenic genes, however this method needed a plant selectable marker gene (SMG) for regenerating events (Hoerster et al., 2020). Here we report an approach that allows excision of both the morphogenic gene and the SMG used in transformation at the same time. As an added benefit this method eliminates any adverse effect from the non-trait (e.g. SMGs) genes in commercial products.

Several strategies have been developed to remove SMGs following plant transformation. One approach is co-transformation with two constructs, one with the SMG and one with the gene of interest. In a transgenic plant with independent insertions of each of these constructs, the selectable marker can be segregated genetically (Hare and Chua, 2002; Puchta, 2003; Darbani et al., 2007; Ling et al., 2016). Alternatively, SMGs can be removed by excision *via* homologous recombination (Puchta, 2000; Zubko et al., 2000), elimination by transposition (Maeser and Kahmann, 1991; Gao et al., 2015) or, by the use of recombinases to excise unwanted DNA. Several recombination systems have been used to excise SMGs, including *Cre/lox* from bacteriophage P1 (Hoess et al., 1982; Hoess and Abremski, 1985), *Flp/frt* from *Saccharomyces cerevisiae* (Cox, 1983; Senecoff et al., 1985), R/RS from *Zygosaccharomyces rouxii* (Araki et al., 1985), and *Gin*/*gix* from bacteriophage (Klippel et al., 1988). Recombinases have been delivered *via* retransformation (Odell et al., 1990; Dale and Ow, 1991), sexual crosses (Bayley et al., 1992; Kilby et al., 1995; Kerbach et al., 2005), or transient expression (Gleave et al., 1999; Kopertekh et al., 2004; Kopertekh and Schiemann, 2005; Jia et al., 2006). In most of these systems, SMG removal takes place after the T0 generation and requires screening multiple plants. An alternative method is to induce excision prior to T0 regeneration. One approach is to design a construct containing SMG and the recombinase genes that are flanked by recombination sites. This has been referred to as “auto-excision” (Verweire et al., 2007; Moravčíková et al., 2008), and allows generation of SMG-free events. Placing the recombinase under the regulation of an inducible/chemical promoter, an expression system that allowed spatial and temporal control (regulated by external or intrinsic signals) was shown to be faster and less resource-intensive (Chong-Pérez and Angenon, 2013; Yau and Stewart, 2013).

We evaluated different strategies for auto-excision prior to regeneration to recover stable T0 plants free of morphogenic genes and in some cases the SMG as well: 1) an auto-excision system using developmentally regulated promoters, 2) an excision-activated marker gene system, and 3) an auto-excision system using an inducible promoter. These excision strategies were evaluated for 1) high transformation frequency, 2) quality event (QE, single-copy of T-DNA, backbone and morphogenic gene free) frequency, 3) ability to generate marker-free T0 plants, and 4) applicability to multiple elite maize inbreds. The use of developmentally regulated promoters driving *Cre* enabled auto-excision of morphogenic genes but resulted in low transformation frequency and QE recovery. These limitations were addressed using heat-shock inducible promoters driving expression of *Cre*, that resulted in higher frequencies of T0 transformation, gene-excision and QE recovery.

## Materials and Methods

### Plant Material

Pioneer temperate maize inbreds (HC69, PH2RT, PH85E, and PH84Z) were used in this study. All plants used for source immature embryos were grown in the greenhouse. One of the inbred lines (HC69) is nonproprietary and publicly available. The other three inbred lines described here are proprietary (PH2RT, PH85E, and PH84Z). In order to protect Corteva Agriscience proprietary germplasm, such germplasm will not be made available except at the discretion of Corteva Agriscience and then only in accordance with all applicable governmental regulations.

### Donor Material and Tissue Culture

Seeds were germinated and grown in a greenhouse at temperature set-points of 25.5/20.0°C (day/night), and 16 h daylight. After 21 d, seedlings were transplanted into 5.9 L pots containing a soil-less substrate composed of 38% Canadian sphagnum peat, 51% composted bark, 8% perlite, and 3% vermiculite by volume and adjusted with lime to a pH of 6.0. Maize ears from the Pioneer inbred lines HC69, PH2RT, PH84Z, and PH85E were collected from the greenhouse (Johnston, Iowa) at 10 to 11 d after pollination, when the immature embryos were 1.5–2.0 mm in length. Ears were sterilized with 20% Clorox (final sodium hypochlorite concentration of 1.65%) for 15 min and rinsed three times with sterile distilled water.

### Culture Media Used for Transformations and Plant Regeneration

Briefly, maize immature embryos (1.5–2 mm) were harvested and used for *Agrobacterium*-mediated transformation, using the media, selection and regeneration methods described previously (Lowe et al., 2018; Chu et al., 2019; Hoerster et al., 2020). For selection, 0.1 mg/L imazapyr was supplemented to somatic embryo formation medium or 150 mg/L G418 was substituted for imazapyr.

### *Agrobacterium*-Mediated Transformation

**Figure 1:**
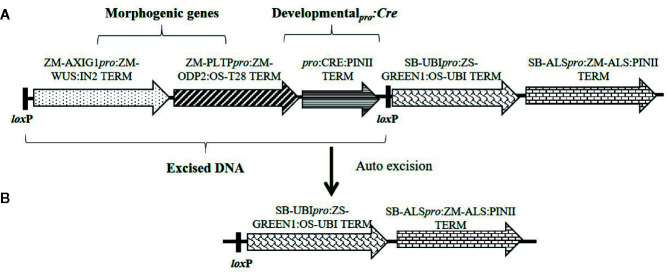
Schematic representation of an auto-excision construct design used for testing different developmentally regulated or stress-inducible promoters to achieve excision of morphogenic genes. **(A)** The excision construct with different promoter combinations driving *Cre* expression (represented by *pro:*CRE) and the DNA fragment to be excised flanked by two directly oriented *lox*P recombination sites. **(B)** The excised product following auto-excision. Refer to **Table S-1** for description of construct components used in T-DNA construction.

**Figure 2:**
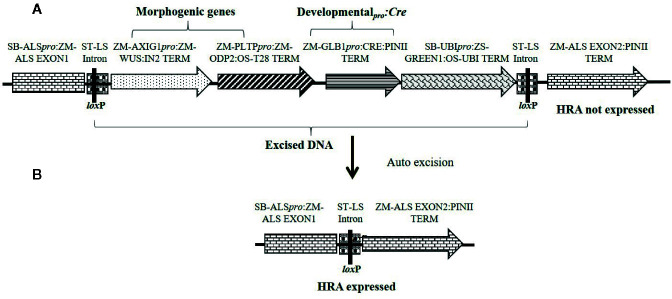
Schematic representation of an auto-excision construct design used for testing developmentally regulated promoters driving *Cre* expression (represented by *pro:*CRE) for excision-activated SMG expression. **(A)** An excision-activated selectable marker construct design with the DNA fragment to be excised flanked by two directly oriented *lox*P recombination sites. **(B)** Following excision, the *HRA* gene is activated and events are selected on a media supplemented with 0.1 mg/L imazapyr. Refer to **Table S1** for description of construct components used in T-DNA construction.

**Figure 3:**
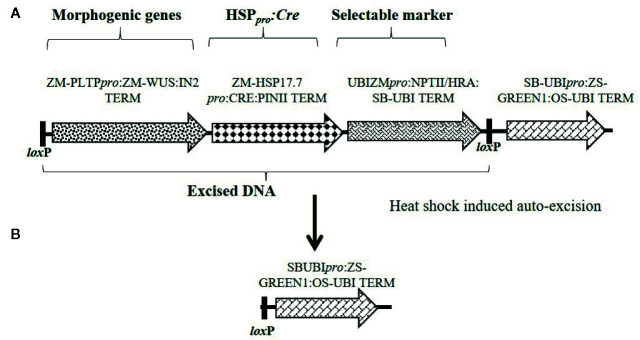
Schematic representation of an auto-excision construct design used for testing elimination of a morphogenic gene and a marker gene using heat shock promoter driving *Cre* expression for controlled gene excision. **(A)** Construct design depicting the order of cassettes including morphogenic genes, *Hsp17.7_pro_* driving *Cre* expression, and the selectable marker (*HRA* or *NPTII*) flanked by directly oriented *lox*P sites (a) which will be excised upon *Cre* expression. **(B)** Following excision, the DNA piece containing the ZS-GREEN expression cassette is left in the T0 event for visual confirmation of excision. Refer to **Table S-1** for description of construct components used in T-DNA construction.

Constructs used in these experiments are illustrated in **Figures 1**–**3** and the individual expression components such as promoters, structural genes and terminators are listed in **Table S1**. The materials reported in this article contain selectable markers (*HRA* and *NPTII*) and reporter genes (*ZS-Green* and *Zs-Yellow*) owned by third parties. Authors may not be able to provide materials including third party genetic elements to the requestor because of certain third-party contractual restrictions placed on the author’s institution. In such cases, the requester will be required to obtain such materials directly from the third party. The authors and authors’ institution do not make any express or implied permission(s) to the requester to make, use, sell, offer for sale, or import third party proprietary materials.

All transformations were done using the thymidine auxotrophic *Agrobacterium tumefaciens* strain LBA4404 THY- containing the pVIR helper plasmid, pPHP71539 (Anand et al., 2018) at OD_550_ of 0.5. The conditions for *Agrobacterium* suspension culture preparation following embryo isolation and infection have been previously described (Lowe et al., 2018; Hoerster et al., 2020). Two selectable markers were used in experiments: *HRA* (Green et al., 2009), a sulfonylurea herbicide resistance marker, driven by the sorghum *Als* promoter for selection with 0.1 mg/L imazapyr in culture medium, or the *Ubi_pro_*:*NPTII* gene for selection with 150 mg/L G418 in culture medium.

### Excision Conditions

**Figure 4:**
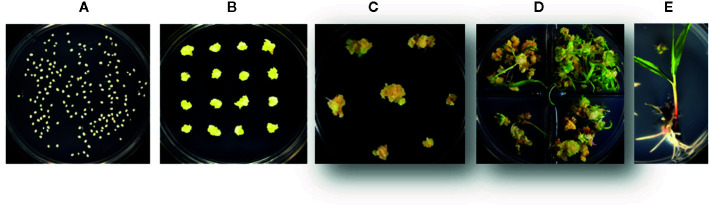
The different stages in rapid maize transformation and heat shock treatment. **(A)** immature zygotic embryos are isolated and infected with *Agrobacterium tumefaciens*, (B) transgenic somatic embryos are placed for 3 weeks on selection media based on selectable marker used (*HRA* or *NPTII*), **(C)** somatic embryos are heat shock treated and transferred to maturation media, **(D)** transgenic plants are regenerated with or without selection pressure for 2 weeks and, **(E)** regenerated plants are placed on a rooting media for 2–3 weeks.

For the developmentally regulated *pro:Cre* testing, no optimization was required. These experiments were performed on two inbreds, HC69 and PHR2HT. The previously described maize transformation protocol (Chu et al., 2019; Hoerster et al., 2020) was used with the transformation stage presented in **Figure 4**. After 3 weeks of selection in maturation media supplemented with either 0.1 mg/L imazapyr (*HRA*) or 150 mg/L G418 (*NPTII*), the plates with somatic embryos were subjected to heat shock treatment in a pre-heated incubator at different temperatures with 70% relative humidity for different time periods. Following heat-shock treatment, the plates were immediately returned to regeneration media with or without selectable marker for 2 weeks in the tissue culture chamber. Regenerated plants with healthy roots were transferred to soil. The initial heat shock treatment involved three different conditions: no heat shock (control), heat shock at 37°C for 1 day, or 42°C for 2 h/day for 3 consecutive days. The heat shock condition was further optimized using following treatments, 1) 42°C, 2 h/day for 2 d, 2) 42°C for 24 h, or 3) 45°C for 2 h/day to identify the best condition.

### Molecular Analyses

All molecular analysis and transgene copy number determination methods were previously described (Wu et al., 2014; Lowe et al., 2016; Hoerster et al., 2020). qPCR data was used to confirm recombinase-mediated excision based on the absence of the transgenes flanked by the *lox*P sites, determine the copy number of structural genes outside the excision DNA, and to screen for the presence of *Agrobacterium* binary construct backbone integration. Genomic DNA samples were extracted from a single piece (200 ng) of fresh leaf tissue from each plant (Truett et al., 2000). Non-transgenic maize inbred lines were used as the negative controls. Quantification was based on detection of amplified gene sequences using gene-specific forward and reverse primers, along with the corresponding gene-specific FAM™ or Vic^®^-based MGB fluorogenic probes (Applied Biosystems). The 2− ΔΔCT method (Livak and Schmittgen, 2001) was used to estimate copy number. Events which are single copy for all the transgenes and excised was used to calculate the excision frequency. Events which were excised with a single copy (SC) of all the transgenes without vector backbone integration were defined as a QE. The usable event (UE) frequency was calculated as transformation frequency times QE frequency. To determine intactness of the excised T-DNA at the insertion site, fifteen QE T0 plants were selected and subjected to Southern-by-Sequencing (Zastrow-Hayes et al., 2015).

Data collected from different experiments were analyzed separately by analysis of variance (ANOVA), with mean separation by LSD (P = 0.05) using JMP Pro 12.2.0 Statistical Discovery software package (SAS Institute Inc., Cary, NC).

## Results

### Excision *via* Developmentally-Regulated Promoters

The presence of morphogenic genes in transgenic events is undesirable because of unpredictable phenotypes (Lowe et al., 2016). Auto-excision of morphogenic genes occurs early in the transformation process which enables trait evaluation in T0 generation and reduces attrition due to T0 sterility. We evaluated several auto-excision designs, using *Cre* driven by various promoters. These included seven different developmentally regulated (embryo or meristem) promoters, the constitutive maize ubiquitin (*ZM-Ubi*) promoter, and the *Agrobacterium* nopaline synthase (*Nos*) promoter (**Table 1**). To facilitate excision, the morphogenic genes (*Wus2* and *Bbm*) and the *Cre* gene cassette were flanked with a single pair of directly oriented *lox*P sites (**Figure 1A**). Following excision morphogenic-gene free events were regenerated as seen in **Figure 1B**. We evaluated two different inbreds (HC69 and PH2RT) to identify *pro*:*Cre* combinations that produced high frequencies of both transformation and excision. Molecular event data is presented in **Table 2**. All constructs tested produced stable transgenic events with some number of properly excised events. The *Ole_pro_ : Cre* had the highest transformation frequencies (27.2–37.1%), while the *Glb1_pro_*:*Cre* construct produced events with higher QE frequencies (8.6–18.4%).

**Table 1:** List of the promoters, their source, and their expression pattern in plants.

Promoters	Source	Expression	Reference
*Kn1*	Maize	Apical Meristem	Gen bank AY312169
*Lec1*	Maize	Early Embryo	(Shane, 2007)
*End2*	Maize	Early Embryo	(Casper et al., 2005)
*Ltp2*	Maize	Early Embryo	(Kalla et al., 1994)
*Glb1*	Maize	Late Embryo	(Liu et al., 1998)
*Ole*	Maize	Late Embryo	(Anand et al., 2017b)
*Rab17*	Maize	Late Embryo/Stress	(Busk et al., 1997)
*Nos*	*Agrobacterium tumefaciens*	Constitutive	(An, 1986)
*Ubi_pro_*	Maize	Constitutive	(Christensen et al., 1992)
*Hsp17.7*	Maize	Heat shock inducible	(Anand et al., 2017a)
*Hsp26*	Maize	Heat shock inducible	(Anand et al., 2017a)
*Rab21*	*Seteria itallica*	Drought inducible	Previously unpublished Corteva Agriscience sequence Si026926m
*Drp12*	*Brachypodium distachyon*	Drought inducible	Previously unpublished Corteva Agriscience sequence Bradi3g43870.1
*Drp1*	*Brachypodium distachyon*	Drought inducible	Previously unpublished Corteva Agriscience sequence Bradi1g37410.1

**Table 2:** Transformation results with different developmentally regulated promoters driving *Cre* expression for auto-excision of morphogenic genes using construct design described in **Figure 1**.

Inbred	Promoter	Embryos transformed	T0 plants	T0 transformation frequency (% ± SE)	Total Single copy events	Excised single copy, backbone-free events	Excision frequency (%)	Quality event (%)	Usable events (%)
PH2RT	*Ltp2*	229	75	32.8 (2.2)^a^	20	10	50.0	13.3	4.4
	*Ole*	228	59	27.2 (3.3)^ab^	20	8	40.0	13.6	3.5
	*Glb1*	280	38	13.6 (1.4)^c^	12	7	58.3	18.4	2.5
	*End2*	174	39	22.4 (2.6)^b^	3	3	100.0	7.7	1.7
	*Ubi*	440	40	9.1 (1.9)^c^	20	12	60.0	30	2.7
HC69	*Rab17*	121	35	28.9 (2.6)^b^	4	1	25.0	2.9	0.8
	*Ole*	151	49	37.1 (2.1)^a^	8	3	37.5	6.1	2
	*Glb1*	230	58	25.2 (1.8)^b^	13	5	38.5	8.6	2.2
	*End2*	178	48	27.0 (2.4)^b^	1	1	100.0	2.1	0.6
	*Ubi*	202	37	18.3 (1.2)^c^	22	3	13.6	8.1	1.5

Data presents the T0 transformation frequency, qPCR detection of the number of excised events and the quality event frequency in two different inbreds, PH2RT and HC69.

Data from three independent transformers was used to determine T0 transformation frequency. The quality events (QE) were identified as single copy, backbone-free, and morphogenic gene-free (excised). The excision frequency was determined as the ratio of the number of excised single-copy events relative to the total single-copy events. The number QEs was divided by the total number of events recovered to calculate the QE frequency. The usable event (UE) frequency is a measure of the number of acceptable transgenic events per 100 embryos that was determined as the product of QE frequency and T0 transformation frequency. Mean values followed by the same letter are not statistically different from each other at the significance level of 0.05.

### Excision *via* Marker Gene Activation

Although we achieved auto-excision with all developmentally regulated promoters tested, even for the best construct the usable events rate was around 2% and 80–90% of events were not excised quality events. To improve efficiency, we designed constructs in which the SMG was activated only upon excision of the morphogenic genes. This approach selected directly for excised events and was expected to increase QE frequency. A similar construct design was previously used to optimize tissue culture conditions for recovering high quality maize transgenic events (Chu et al., 2019). A schematic design of the construct is depicted in **Figure 2A** and the quality excised product in **Figure 2B**. For these experiments, either the *Glb1* or the *Ole *promoters were used to drive *Cre* expression. The data from side-by-side testing of these two promoters using the construct design described in **Figure 2** are summarized in **Table 3**. The construct containing *Glb1_pro_*:*Cre* improved T0 transformation and QE frequencies (1.8 and 1.4-fold), compared to the developmentally regulated gene-excision approach. When *Ole_pro_ : Cre* was used, the T0 transformation frequency was similar (>1.1-fold) while the QE frequency increased approximately 1.7-fold. The excision frequency was higher when excision-activated selection was used, with excision frequencies of 53.3 (*Ole_pro_ : Cre*) and 68.4% (*Glb_pro_ : Cre*) when compared to the previous approach. Additionally, no null events (escapes) were identified by qPCR analysis.

**Table 3:** Transformation results from excision-activated marker gene selection using either the *Glb1_pro_* or the *Ole_pro_* driving *Cre* expression using construct design described in **Figure 2**.

Promoter	Embryostransformed	T0 plants	T0 transformation frequency (% ± SE)	Total single copy events	Excised single copy, backbone-free events	Excision frequency (%)	Quality event (%)	Usable events (%)
*Glb1*	126	57	44.7 (2.8)^a^	19	13	68.4	13.3	5.6
*Ole*	112	45	40.2 (1.9)^a^	15	8	53.3	8.8	3.6

Data from two independent transformers was used to determine T0 transformation frequency. Quality events (QE) were identified as single copy, backbone-free, and morphogenic gene-free (excised). The excision frequency was determined as the ratio of the number of excised single-copy events relative to the total single-copy events. The number QEs was divided by the total number of events recovered to calculate the QE frequency. The usable event (UE) frequency is a measure of the number of acceptable transgenic events per 100 embryos that was determined as the product of QE frequency and transformation frequency. Mean values followed by the same letter are not statistically different from each other at the significance level of 0.05.

Data presents the T0 transformation frequency, qPCR detection of the number of excised events and the quality event frequency in maize inbred HC69.

The *Glb_pro_ : Cre* construct design was further evaluated in two additional inbreds, PH84Z and PH85E, alongside HC69 for comparison (**Table 4**). QEs were recovered in all three inbreds, which were free of the morphogenic genes with no escapes. Excision frequency was similar (55–58%) across all the inbreds; QE frequencies varied by genotype: 8.7 (HC69), 27.7 (PH85E), and 6.7% (PH84Z) leading to differences in usable quality event frequency (UE, quality events per 100 embryos): 4.3 (HC69), 3.6 (PH85E), and 1.9% (PH84Z).

**Table 4:** Transformation results from excision-activated marker gene selection using Glbpro driving Cre expression using construct design described in **Figure 2**.

Inbred	Embryos transformed	T0 plants	T0 transformation (% ± SE)	Total Single copy events	Excised single copy, backbone-free events	Excision frequency (%)	Quality event (%)	Usable events (%)
HC69	393	196	49.9 (3.9)^a^	31	17	54.8	8.7	4.3
PH85E	363	47	12.9 (1.3)^c^	22	13	59.0	27.7	3.6
PH84Z	367	105	28.6 (2.5)^b^	12	7	58.3	6.7	1.9

Data from two independent transformers was used to determine T0 transformation frequency. The quality events (QE) were identified as single copy, backbone-free, and morphogenic gene-free (excised). The excision frequency was determined as the ratio of the number of excised single-copy events relative to the total single-copy events. The number QEs was divided by the total number of events recovered to calculate the QE frequency. The usable event (UE) frequency is a measure of the number of acceptable transgenic events per 100 embryos that was determined as the product of QE frequency and transformation frequency. Mean values followed by the same letter are not statistically different from each other at the significance level of 0.05.

Data presents the T0 transformation frequency, qPCR detection of the number of excised events and the quality event frequency in three maize inbreds (HC69, PH85E, and PH84Z).

### Excision *via* Stress-Inducible Promoters

To further improve efficiency, a series of stress-inducible promoters were tested for excision of morphogenic genes. The promoters were selected from a set of genes induced by heat (maize *Hsp17.7* and *Hsp26)* and drought (*ZmRab17*, *SiRAB21*, *BdDRP1*, and *BdDRP12*). The construct design is identical to that described in **Figure 1**, where stress-inducible promoters drive *Cre* expression as represented by *pro*:*Cre*. In preliminary screening, embryos derived from HC69 were infected with an *Agrobacterium* strain containing one of the six constructs and, subsequently subjected to one of three different conditions: no heat shock (control), heat shock at 37°C for 1 day, or 42°C for 2 h/day for 3 consecutive days. The different steps in maize immature embryo transformation process included embryo infection with *Agrobacterium* strain containing the construct (**Figure 4A**), selection of transgenic events on media supplemented with selectable marker for 3 weeks (**Figure 4B**), heat-shock treatment step (**Figure 4C**) followed by immediate transfer to regeneration media with selection pressure (**Figure 4D**) and rooting (**Figure 4E**), before the events were sent to greenhouse. The auto-excision frequencies under induced and non-induced conditions were determined by qPCR analysis. Somatic embryos on maturation media (18 dpi) with 0.1 mg/L imazapyr were subjected to one of the heat conditions and moved onto a rooting media with 0.1 mg/L imazapyr following heat shock (**Figure 4C**).

All promoters except *Hsp26* were leaky under non-induced conditions, resulting in gene-excision rates from 3.4 (*Rab17_pro_*) to 36% (*BdRab21_pro_*) compared to zero in the *Cre*-minus construct (**Table 5**). For a subset of the promoters (* HSP 17.7*, *Hsp26*, *Drp1*, and *Drp12*), higher excision frequencies ranging from 43 to 100%, were observed in the 42°C, 2 h/day for 3 days heat treatment. Longer exposure of the somatic embryos at 37°C adversely effected T0 event recovery, compared to a short pulse of heat shock at 42°C (2 h/day for 3 days). Based on the recovery of excised T0 events with *Hsp26_pro_* construct at 42°C treatment compared to 37°C treatment, this promoter appeared to be induced only at higher temperatures.

**Table 5:** Transformation results from screening of six different inducible promoters driving *Cre* expression for controlled gene excision.

Promoter	Control					37°C, 1 day					42°C, 2 h/day for 3 days				
	Embryos	T0 plants	Single copy event (number)	QE	Excision frequency	Embryos	T0 plants	Single copy event (number)	QE	Excision frequency (%)	Embryos	T0 plants	Single copy event (number)	QE	Excision frequency (%)
					(%)										
*Hsp17.7*	455	59	18	5	27.8	50	6	3	2	66.7	50	20	4	4	100
*Hsp26*	450	98	0	0	0	50	5	0	0	0	50	21	7	3	43
*Rab17*	455	127	29	1	3.4	50	10	0	0	0	50	18	0	0	0
*Rab21*	455	101	22	8	36.4	50	13	1	1	100	50	20	0	0	0
*Drp12*	450	79	18	2	11.1	50	16	0	0	0	50	22	3	2	66.7
*Drp1*	438	90	29	8	27.6	50	8	0	0	0	50	27	11	5	45.5
Control (no *Cre*)	450	182	0	0											

Data from two independent transformers was used to determine T0 transformation frequency. The quality events (QE) were identified as single copy, backbone-free, and morphogenic gene-free (excised). The excision frequency was determined as the ratio of the number of excised single-copy events relative to the total single-copy events.

For this study, three different conditions were evaluated: two heat shock treatments (37°C for 1 day and 42°C, 2 h/day for 3 consecutive days) and no heat (control). Data presents the qPCR detection of the number of excised events and excision frequency across the different promoters, and a control construct without the Cre gene, in maize inbred HC69.

Additional experiments were performed to further evaluate gene excision and optimize heat shock conditions using three of the inducible promoters (*Hsp17.7*, *Hsp26*, and *Drp12*). HC69 embryos infected with the three constructs were subjected to heat shock treatment at the maturation stage (**Figure 4C**). One of three different treatments were applied 1) no heat shock (control), 2) 42°C for 2 h, and 3) 42°C, 2 h on 3 consecutive days to determine frequencies of excision and UE recovery (**Table 6**). Consistent with the previous observation, *Hsp17.7_pro_* driving *Cre* expression under both heat treatments resulted in higher excision rates (62.5–69.2%) producing higher UE rates (10 to 18%) compared to *Hsp26 _pro_* and *Drp12 _pro_*. Based on the data, we identified *Hsp17.7_pro_* as the preferred promoter for auto-excision with heat shock of 42°C for 2 h.

**Table 6:** Transformation results optimizing the heat shock conditions for controlled gene excision using three inducible promoters driving *Cre* expression.

Promoter	Control						42°C, 2 h						42°C, 2 h/day for 3 days					
	Embryos	T0 Plants	SC (number)	QE	Excision frequency	UE%	Embryos	T0 plants	SC (number)	QE	Excision frequency	UE	Embryos	T0 plants	SC (number)	QE	Excision frequency	UE (%)
					(%)						(%)	(%)					(%)	
*Hsp17.7*	50	18	8	1	12.5	2	50	17	8	5	62.5	10	50	15	13	9	69.2	18
*Hsp26*	50	18	0	0	0	0	50	21	5	2	40	4	50	9	3	2	66.7	4
*Drp12*	50	11	4	1	25	2	50	9	2	1	50	2	50	14	5	1	20	2

Data from two independent transformers was used to determine T0 transformation frequency. The quality events (QE) were identified as single copy, backbone-free, and morphogenic gene-free (excised). The excision frequency was determined as the ratio of the number of excised single-copy events (SC) relative to the total single-copy events. The number QEs was divided by the total number of events recovered to calculate the QE frequency. The usable event (UE) frequency is a measure of the number of acceptable transgenic events per 100 embryos that was determined as the product of QE frequency and transformation frequency. Mean values followed by the same letter are not statistically different from each other at the significance level of 0.05.

The three different conditions evaluated were: no heat (control) and two heat shock treatments (42°C for 2 h and 42°C, 2 h/day for 3 consecutive days). The data presents the qPCR detection of the number of excised events and excision frequency across the different promoters in the study as compared to a control construct without the Cre gene in maize inbred HC69.

### Optimization of Heat-Shock Conditions to Improve Auto-Excision

Further experiments were designed with *Hsp17.7_pro_* and *Hsp26_pro_* to optimize excision conditions. After three weeks of selection, somatic embryos at the maturation stage were subjected to one of three different heat conditions 1) 42°C, 2 h/day for 2 d, 2) 42°C for 24 h, or 3) 45°C for 2 h/day to determine frequencies of excision and UE. Across the treatments, transformation frequencies ranged from 35–54.9%, except in the 42°C for 24 h treatment of embryos with *Hsp17.7_pro_* driving *Cre* expression, which was lower (**Table 7**). The heat treatments increased excision rates, which varied with the conditions applied. Of the two *Hsp* promoters tested, *Hsp17.7_pro_* resulted in events with higher excision frequency (75% at 42°C for 24 h and 77.8% at 45°C for 2 h) compared to *Hsp26_pro_* (66.7 and 61.9%). The 45°C/2 h treatment worked worked best for both *Hsp* promoters.

**Table 7:** Optimizing heat shock conditions for controlled gene excision using heat shock promoters *Hsp17.7* and *Hsp26* driving *Cre* expression.

Promoter	Treatments	Embryos transformed	T0 plants	T0 transformation (% ± SE)	Single copy events (numbers)	Quality events(numbers)	Excision frequency (%)	Usable event (%)
*Hsp17.7*	none	102	56	54.9 (4.4)^a^	18	6	33.3	5.9
	42°C, 2 h/d, 2 d	102	39	38.2 (2.1)^b^	16	9	56.2	8.8
	42°C/24 h	102	16	15.7 (1.8)^c^	8	6	75	5.9
	45°C/2 h	102	50	49.0 (3.2)^a^	18	14	77.8	13.7
*Hsp26*	none	100	53	53.0 (4.0)^a^	18	1	5.6	1
	42°C, 2 h/d, 2 d	100	35	35.0 (1.2)^b^	18	12	66.7	12
	42°C/24 h	100	41	41.0 (2.2)^b^	15	10	66.7	10
	45°C/2 h	100	50	50.0 (2.3)^a^	21	13	61.9	13

The heat treatments increased excision rates, which varied with the conditions applied. Of the two Hsp promoters tested, Hsp17.7_pro_ resulted in events with higher excision frequency (75% at 42°C for 24 h and 76.6% at 45°C for 2 h) compared to Hsp26_pro_ (66.7 and 61.9%). The treatment, 45°C for 2 h worked best for both Hsp promoters.

Four different conditions were evaluated side-by-side using split ears including no heat (control) and three heat shock treatments (42°C, 2 h/d for 2d; 42°C/24 h; and 45°C/2 h). Transformation results and qPCR detection of the number of excised quality events, frequencies of excision and usable event are presented.

Data from two independent transformers was used to determine T0 transformation frequency. The quality events (QE) were identified as single copy, backbone-free, and morphogenic gene-free (excised). The excision frequency was determined as the ratio of the number of excised single-copy events relative to the total single-copy events. The number QEs was divided by the total number of events recovered to calculate the QE frequency. The usable event (UE) frequency is a measure of the number of acceptable transgenic events per 100 embryos that was determined as the product of QE frequency and transformation frequency. Mean values followed by the same letter are not statistically different from each other at the significance level of 0.05.

### Concurrent Elimination of Morphogenic and Plant Selectable Marker Genes

Next, we developed a strategy that simultaneously excised both the morphogenic genes and the SMG. Two different SMGs, *HRA*, and *NPTII* were tested. The construct design was slightly changed to enable excision of the SMG by including it as part of the excised DNA (morphogenic genes and *Cre)* flanked with a single pair of directly oriented *lox*P sites (**Figure 3A**) and the resulting excised events are free of SMG (**Figure 3B**). The binary construct designs with different selectable marker, morphogenic gene and a reporter gene *Zs-GREEN* is illustrated in **Figure 3A**. Following transformation and selection (either 0.1 mg/L imazapyr for the *HRA* gene or 150 mg/L G418 for the *NPTII* gene), the somatic embryos were heat-shock treated at 45°C for 2 h and moved into selection free regeneration and rooting media. Transformation data are presented in **Table 8**. Both *HRA* and *NPTII* constructs produced T0 plants free of morphogenic genes and SMG in the three inbreds tested. With the *HRA* construct, lower frequencies of QEs and UEs were observed and 2-fold more null events were produced compared to the *NPTII* construct. The excision frequency was comparable in both *HRA* and *NPTII* constructs. Irrespective of the differences, both selectable markers produced high frequencies of single copy, backbone-free events which are free of the morphogenic and marker genes.

**Table 8:** Transformation results and molecular event data using the *Hsp17.7* heat shock promoter for controlled excision of both morphogenic gene and marker gene in three maize inbreds (HC69, PH85E, and PH84Z).

Inbred	Selectable marker	Embryos transformed (number)	T0 plants (number)	T0 transformation (%)	Total Single copy events	Quality event (number)	Quality events (%)	Excision frequency (%)	Usable event (%)	Null (%)
HC69	*NPTII*	315	200	63.5	53	46	23	87	14.6	17.1
	*HRA*	407	281	69	55	45	16	82	11.1	37.3
PH85E	*NPTII*	219	64	29.2	24	23	35.9	96	10.5	15.3
	*HRA*	320	124	38.8	32	31	25	97	9.7	42.5
PH84Z	*NPTII*	356	145	40.7	38	19	13.1	50	5.3	14.2
	*HRA*	365	169	46.3	23	14	8.3	61	3.8	41.8

Data from two independent transformers was used to determine T0 transformation frequency. The quality events (QE) were identified as single copy, backbone-free, morphogenic and marker gene-free (excised). The number of QEs was divided by the total number of events analyzed to calculate the QE frequency. The excision frequency was determined as the ratio of the number of excised single-copy events relative to the total single-copy events. The usable event (UE) frequency is a measure of the number of acceptable transgenic events per 100 embryos that was determined as the product of QE frequency and transformation frequency.

Two different SMGs were evaluated, HRA (resistance to the sulfonylurea herbicide ethametsulfuron) and NPTII (resistance to antibiotic G418), using the same construct design with the same set of morphogenic genes as shown in **Figure 3**. Transformation results and qPCR detection of the number of excised quality events, frequencies of excision and usable event are presented.

To confirm the intactness of T-DNA following excision, 15 T0 quality events (five T0 plant per inbred, **Table 8**) transformed with the *NPTII* construct were analyzed with Southern-by-sequencing analysis (SbS, Zastrow-Hayes et al., 2015). SbS utilizes capture-based target enrichment of samples prior to next-generation sequencing (NGS) and is used to determine the insertion sequence and intactness of the inserted DNA at the insertion site. Twelve out of the fifteen T0 plants had intact single copy T-DNA insertion, free of morphogenic and marker genes. The other three plants failed SbS, two because they contained truncations of the T-DNA and one because an additional T-DNA fragment was inserted at a different location.

### Progeny Analysis

**Figure 5:**
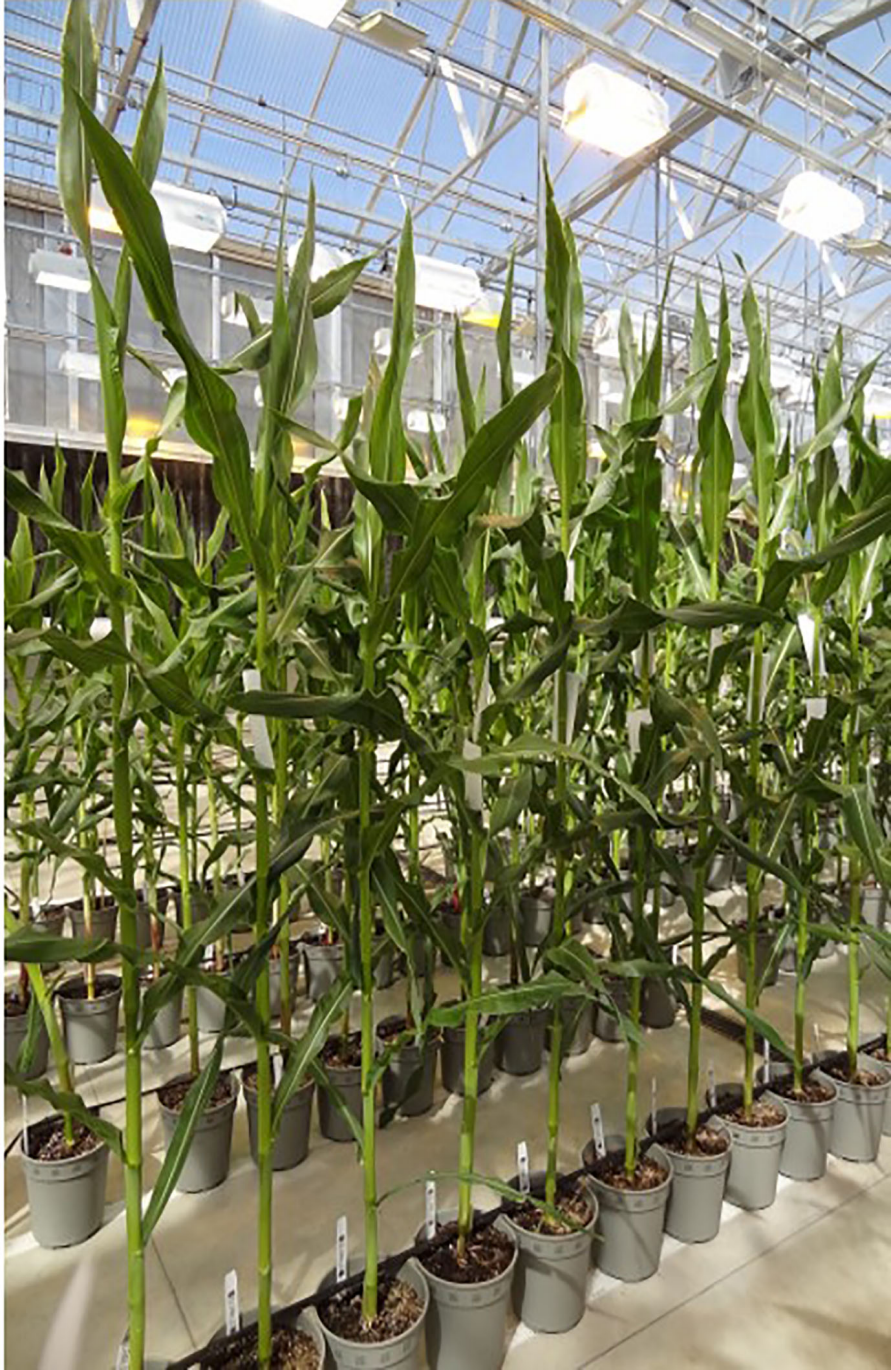
The T1 plants of PHR84Z. Homozygous T1 plants from PH84Z which were determined to be quality events, free of marker and morphogenic genes exhibited normal plant phenotype.

To study the inheritance and segregation of the morphogenic and SMG-free events, we screened single-copy T0 plants free of morphogenic gene and SMG produced from the *NPTII* construct. Thirteen T0 QE plants, six plants from HC69, and seven plants from PHR84Z, were selected for progeny analysis. These plants were self-pollinated and all 13 events produced seeds. T1 plants were evaluated for zygosity using qPCR to evaluate copy number of *Cre* and *NPTII* genes (excised DNA). Twelve of the 13 events showed the expected Mendelian inheritance of a single copy T-DNA integration (1:2:1; chi-square p-value >0.05) in the T1 generation (**Table 9**). All T1 plants looked normal (**Figure 5**) and set seeds.

**Table 9:** Observed and expected number of homozygous, hemizygous, and null plants for T-DNA integration copy number in in T1 generation of 13 SC excised quality events across two maize inbreds (PH84Z and HC69).

Inbred	Event ID	Total Plants	Homozygous	Hemizygous	Null	Chi-square	P-value*
PH84Z	ZMYF66.001.83A	23	7	11	5	0.39	0.82
	ZMCJK9.001.74A	31	10	13	8	0.76	0.68
	ZMCJK9.001.13A	30	8	18	4	2.03	0.36
	ZMCJK9.001.96A	32	6	17	9	0.69	0.71
	ZMCJK9.001.34A	30	10	12	8	1.5	0.47
	ZMCJK9.001.77A	24	5	10	9	2	0.36
	ZMCJK9.001.3A	27	4	17	6	2.07	0.35
HC69	ZMNW4W.001.72A	23	11	7	5	6.65	0.03
	ZMNW4W.001.30A	31	11	13	7	1.83	0.39
	ZMNW32.001.49A	32	4	17	11	3.19	0.2
	ZMNW32.001.58A	31	8	14	9	0.35	0.84
	ZMNW32.001.43A	32	9	10	13	5.5	0.06
	ZMNW32.001.65A	32	9	14	9	0.5	0.78

*No statistically significant deviations identified from expected 1:2:1 (homozygous:hemizygous:null) segregation at 5% level.

## Discussion

In maize, direct induction of somatic embryos capable of rapidly germinating from immature embryos (without a callus phase) has been demonstrated using the auxin-inducible promoter *Axig1* driving *Wus2* expression in combination with *Bbm* driven by a maize *PLTP* promoter (Lowe et al., 2018). However, the presence of morphogenic genes in regenerated plants led to abnormal phenotypes (Lowe et al., 2016). Therefore, removing morphogenic genes is imperative for accurate event evaluation and product development. We achieved this by developing a gene excision system. Morphogenic gene excision was accomplished using a drought-inducible *Rab17* promoter driving *Cre* recombinase expression (Vilardell et al., 1991). Although this approach worked, the desiccation step reduced event recovery and not all event achieved excision (Lowe et al., 2016).

In order to develop a more efficient systems, promoters of seven developmentally regulated genes, the *Knotted-1* (*Kn1*) (Bolduc et al., 2012), *Leafy cotyledon1* (*Lec1*) (Pelletier et al., 2017), barley *Lipid transfer protein2* (*Ltp2*) (Kalla et al., 1994), an early embryo response gene (*End2*) (Casper et al., 2005), *Globulin1* (*Glb1*) (Belanger and Kriz, 1991), and *Olesin* (*Ole*) (Anand et al., 2017b) were evaluated for their ability to express *Cre* and in order to excise morphogenic genes. Unlike inducible promoters these developmentally regulated promoters do not require physical or chemical induction. Morphogenic gene removal was observed using developmentally regulated promoters, but with lower QE frequencies. This may be attributable to premature expression of *Cre*, leading to untimely excision of the developmental genes.

We developed a method for regenerating events that are free of morphogenic genes using an excision-activated marker gene approach (Chu et al., 2019). Developmentally regulated promoters *Glb1* and *Ole* that are active during late embryo development (Kriz et al., 1990; Anand et al., 2017b), were used to express *Cre*, leading to auto-excision. After excision, the HRA gene is activated (Chu et al., 2019). This strategy improved event recovery, however a large proportion of T0 events were multi-copy and non-excised. Rapid maize transformation was previously shown to produce high frequency of multi-copy events (Lowe et al., 2018) and our data is consistent with the observation. Another possibility is the restricted activation of the developmental promoters leading to partial/incomplete excision, which does not work in rapid maize transformation for enriching quality events.

Inducible promoters can be used to regulate expression of recombinase genes. These inducible promoters predominantly fall into two categories; 1) heat shock- or stress-inducible promoters (Kilby et al., 1995; Cuellar et al., 2006; Zhang et al., 2006; Du et al., 2019), and 2) chemical inducible promoters (Gatz, 1996; Zuo and Chua, 2000). Among the stress-inducible promoters tested, *Hsp17.7_pro_* and *Hsp26_pro_* worked best based on a higher frequency of T0 transformation, gene excision and UE rate. In maize, the regulation of *Hsp* promoters in response to stresses has been described (Pegoraro et al., 2011), including accumulation of *Hsp* proteins under temperatures over 32–33°C (Ristic et al., 1991; Vierling, 1991) and enhanced *Hsp70* synthesis under drought and/or heat (Hu et al., 2010). The heat-inducible auto-excision system was previously described using a construct design that involves *Hsp70_pro_* driving the *Cre* recombinase for elimination of the SMG (egfp) while a second marker gene, expressing the anthocyanin pigmentation (Rsc) gene, was used for event sorting (Du et al., 2019). While successful, the strategy has limited practical application requiring tracking of transgenes in the T1 generation and subsequent segregation, which is resource-and time-intensive.

We developed a reliable and efficient method to obtain morphogenic gene-free events at high frequencies (66–77% of the total events generated). The overall objective was to develop an efficient auto-excision system for rapid maize transformation, with the purpose of eliminating both morphogenic and marker genes, that is highly efficient to meet the needs of high throughput maize transformation. The method we developed resulted in the elimination of morphogenic and marker genes at the maturation stage of transformation at high frequencies (ranging from 60–97%) in multiple elite inbreds. This was achieved by optimizing tissue culture conditions, optimization of heat shock treatment and identifying a versatile SMG. The stably transformed plants were normal, produced seeds and showed stable transmission of the integrated T-DNA to the next generation.

## Conclusion

Our objective is to remove morphogenic and selectable marker genes from the events generated from rapid maize transformation. The first generation of rapid maize transformation method was designed to improve the transformation rates and to extend transformation capabilities to many genotypes. Subsequently, we demonstrated a viable second-generation alternative, using a mixture of an *Agrobacterium* strains, one with non-integrating *Wus2* gene and the other with a combination of structural genes to regenerate transgenic plants free of morphogenic genes. Even though this simplifies vector construction, however, the process still relies on a SMG for recovery of stable transgenic events. This study demonstrated a viable third alternative, relying on inducible promoters for auto-excision of both the morphogenic genes and the SMG in the early stages of maize transformation. The stable transformed plants recovered by this method are free of the morphogenic genes and marker genes, a desirable quality for transgene evaluation and in commercial products.

## Data Availability Statement

The datasets presented in this study can be found in online repositories. The names of the repository/repositories and accession number(s) can be found in the article/**Supplementary Material**.

## Author Contributions 

AA, EW, KL, WG-K, TJ, and NC conceived the research idea. AA, EW, KL, and WG-K designed constructs and research. NW, MA, GH, and LR conducted maize transformation and optimization. EW and AA, performed data analysis. AA, WG-K, TJ, and NC wrote the manuscript.

## Conflict of Interest

NW, MA, GH, LR, EW, KL, WG-K, and AA are inventors on pending applications on this work and a related work are current employees of Corteva Agriscience who owns the pending patent applications. TJ and NC are current employees of Corteva Agriscience.

## Abbreviations

*Bbm*, Babyboom; *Cre*, CRE recombinase; HSP, Heat-shock promoters; SMG, Selectable marker-free; Pro, promoters; QE, Quality events; UE, Usable event; *Wus*2, Wuschel2.
