# Supplementary material for: An Efficient Gene Excision System in Maize
Source: Front Plant Sci. 2020 Sep 2;11:1298. doi: 10.3389/fpls.2020.01298 (PMC7492568; doi:10.3389/fpls.2020.01298)
Supplement: Supplementary file 1 [file Table_1.docx]

Table S-1. Construct components used in T-DNA construction.

| **Component type** | **Label** | **Description** | **References** |
| --- | --- | --- | --- |
|  |  |  |  |
| **Promoters** | *Sb-Als_pro_* | The sorghum ALS promoter | SB-ALS promoter and 5’UTR, DOE-JGI Sbi v3.1, SBChr04, bases 49239164-49240031. DOE-JGI Sbi v3.1 corresponds to Sorghum bicolor BTx623 assembly v3.0.1 and gene annotation v3.1 available from phytozome (http://phytozome.jgi.doe.gov/). Chromosome 4 of Sbi v3.1 is registered as NCBI accessions NC_012873.2 and CM000763.3 |
|  | *Pltp_pro_* | Maize phospholipid transferase promoter | See GenBank sequence (MN380778) |
|  | *Axig1_pro_* | The maize Axig1 promoter | (Garnaat et al., 2002) |
|  | *Sb-Ubipro* | The sorghum Ubiquitin promoter | (Shane, 2007) |
|  |  |  |  |
| **3' Sequences** | *In2-2* | The maize IN2-2 terminator | (Hershey and Stoner, 1991) |
|  | *PINII* | The potato proteinase inhibitor II (pinII) 3’sequence | (An et al., 1989) |
|  | *Os-Ubi 3'* | The rice Ubiquitin terminator | Terminator region of the rice Ubiquitin (Os06g46770.1), unpublished |
|  | *Sb-Ubi 3'* | The sorghum Ubiquitin terminator | (Shane, 2007) |
|  | *Os-T28 3'* | The T28 3' regulatory sequence from *Oryza sativa* | (Bhyri et al., 2014) |
| **Marker genes** | *NPTII* | Maize codon-optimized Neomycin Phosphotransferase II | Previously unpublished Corteva Agriscience sequence |
|  | *HRA* | The maize ALS double mutant gene conferring herbicide resistance | (Green et al., 2009) |
|  | *Zs-YELLOW* | The Zs-Yellow1 N1 gene encoding a yellow fluorescent protein from *Zoanthus sp* | (Matz et al., 1999) |
| **Maize morphogenic genes** | *Zm-Wus2* | The maize *Wuschel2* (*Wus2*) gene | (Lowe et al., 2007) |
|  | *Zm-Bbm* | The maize *Baby boom* gene (*Bbm*) | (Gordon-Kamm et al., 2005) |
| **Recombinase Expression Cassettes** | *Cre* | A maize-optimized *Cre* recombinase gene (originally from the P1 bacteriophage), with an inserted potato LS1 intron | (Odell et al., 1990) |
| **Recombinase Target Sites** | *lox*P | The recombinase target site for the Cre recombinase from *E. coli* | (Odell et al., 1990) |

References

Bhyri, P., Khrishnamurthy, N., Narayanan, E., Nott, A., and Sarangi, R. (2014). *Novel*

*plant terminator sequences*. USPTO. United States patent US 20140130205.

Available at: https://patents.google.com/patent/US20140130205A1/en

Garnaat, C., Lowe, K. S., and Roth, B. A. (2002). *Zm-AXIG1-specific*

*polynucleotides and methods of use*. International Patent WO2002006499A2.

Available at: https://patents.google.com/patent/WO2002006499A2/en

Gordon-Kamm, W. J., Helentjaris, T., Lowe, K., Shen, B., Tarczynski, M., and

Zheng, P. (2005). *Ap2 domain transcription factor Odp2 (ovule development*

*protein 2) and methods of use*. International patent. International patent

WO2005075655A2. Available at: https://patents.google.com/patent/

WO2005075655A2

Lowe, K. S., Cahoon, R. E., Scelonge, C. J., Tao, Y., Gordon-Kamm, W. J., Bruce,

W. B., et al. (2007). *Wuschel (WUS) gene homologs*. International patent.

WO2005063990A2. Available at: https://patents.google.com/patent/

WO2005063990A2/en
